# The PEARL score predicts 90-day readmission or death after hospitalisation for acute exacerbation of COPD

**DOI:** 10.1136/thoraxjnl-2016-209298

**Published:** 2017-02-24

**Authors:** C Echevarria, J Steer, K Heslop-Marshall, S C Stenton, P M Hickey, R Hughes, M Wijesinghe, R N Harrison, N Steen, A J Simpson, G J Gibson, S C Bourke

**Affiliations:** 1 North Tyneside General Hospital, North Shields, Newcastle upon Tyne, UK; 2 Institute of Cellular Medicine, Medical School, Newcastle University, Newcastle upon Tyne, UK; 3 Royal Victoria Hospital, Newcastle upon Tyne, UK; 4 Northern General Hospital, Sheffield, South Yorkshire, UK; 5 Royal Cornwall Hospital, Truro, Cornwall, UK; 6 University Hospital of North Tees, Stockton-on-Tees, Cleveland, UK; 7 Institute of Health and Society, Newcastle University, Newcastle upon Tyne, UK

**Keywords:** COPD Exacerbations, COPD epidemiology

## Abstract

**Background:**

One in three patients hospitalised due to acute exacerbation of COPD (AECOPD) is readmitted within 90 days. No tool has been developed specifically in this population to predict readmission or death. Clinicians are unable to identify patients at particular risk, yet resources to prevent readmission are allocated based on clinical judgement.

**Methods:**

In participating hospitals, consecutive admissions of patients with AECOPD were identified by screening wards and reviewing coding records. A tool to predict 90-day readmission or death without readmission was developed in two hospitals (the derivation cohort) and validated in: (a) the same hospitals at a later timeframe (internal validation cohort) and (b) four further UK hospitals (external validation cohort). Performance was compared with ADO, BODEX, CODEX, DOSE and LACE scores.

**Results:**

Of 2417 patients, 936 were readmitted or died within 90 days of discharge. The five independent variables in the final model were: Previous admissions, eMRCD score, Age, Right-sided heart failure and Left-sided heart failure (PEARL). The PEARL score was consistently discriminative and accurate with a c-statistic of 0.73, 0.68 and 0.70 in the derivation, internal validation and external validation cohorts. Higher PEARL scores were associated with a shorter time to readmission.

**Conclusions:**

The PEARL score is a simple tool that can effectively stratify patients' risk of 90-day readmission or death, which could help guide readmission avoidance strategies within the clinical and research setting. It is superior to other scores that have been used in this population.

**Trial registration number:**

UKCRN ID 14214.

**Key messages**

**What is the key question?**

- In patients admitted to hospital with an exacerbation of COPD, what predicts 90-day readmission or death?

**What is the bottom line?**

- The PEARL prognostic score comprises five indices: **P**revious admissions, **e**MRCD score, **A**ge, **R**ight-sided heart failure and **L**eft-sided heart failure, and is a consistent and accurate predictor of 90-day readmission or death.

**Why read on?**

- Currently, postdischarge care is directed by clinical judgement, although clinical judgement alone is suboptimal: the PEARL score is easy to apply at the bedside using indices that are routinely available in all patients, accurately risk stratifies patients according to risk of readmission and may inform postdischarge planning in patients admitted with acute exacerbation of COPD.

## Introduction

Acute exacerbation of COPD (AECOPD) is one of the most common reasons for hospital admission and one-third of patients are readmitted within 90 days.^1^ ^2^ Clinicians are poor at identifying patients at particular risk of readmission.^3^ A simple and accurate prognostic tool would help identify those who may benefit most from additional health and social care services, and this hopefully will translate into improved outcomes for patients and more efficient use of scarce resources. A condition-specific score to predict this outcome following hospitalisation for AECOPD has not been developed.

Within the CODEX study,^4^ the ADO, BODEX, CODEX and DOSE scores were all assessed for their performance at predicting the combined outcome of readmission or death without readmission in patients with COPD (see table 1). However, most were originally developed to predict death (ADO^5^ and BODEX^6^) or health status (DOSE^7^) and all offer only modest prediction of readmission/death without readmission. The performance of CODEX was superior to the updated ADO and BODEX score, with a trend towards better performance than DOSE, but this comparison was in the CODEX derivation population.^4^ Derivation studies bias results in favour of the derived tool, in this instance CODEX, and so comparison in an external validation cohort is required.

**Table 1:** Prognostic scores and their components

	Measured indices											Study population and primary outcome				AUROC for death or readmission^4^ ^8^
	Age	BMI	Comorbidity*	Dyspnoea†	Airflow obstruction‡	Exacerbations§	Severe exacerbations¶	Smoking status	Length of stay	Acuity**	ED visits last 6 months	COPD specific?	Stable-state or exacerbating at inclusion	Outpatients or admitted patients	Primary outcome	
ADO^5^	√			√	√							√	Both	Both	Death	0.58
BODEX^6^		√		√	√		√					√	Stable	Outpatients	Death	0.61
CODEX^4^††	√		√	√	√		√					√	AECOPD	Mostly admitted	Death or readmission	0.67
DOSE^7^				√	√	√		√				√	Unclear	Outpatients	Health status‡‡	0.64
LACE^8^			√						√	√	√	X	N/A	Admitted	Death or readmission	0.68§§

*Charlson Comorbidity Index.

†mMRC dyspnoea score.

‡FEV_1_ %predicted.

§Patient reported AECOPD in previous year.

¶AECOPD in previous year requiring admission or ED attendance.

**Elective or emergency admission.

††Uses age-adjusted Charlson Comorbidity Index.

‡‡Measured by the clinical COPD questionnaire.

§§30-day death or readmission.

AECOPD, acute exacerbation of COPD; AUROC, area under the receiver operating characteristic; BMI, body mass index; ED, emergency department; mMRC, modified Medical Research Council; n/a, not available.

LACE^8^ was primarily derived to assess readmission/death without readmission in unselected emergency and elective admissions, and has not been compared with the other scores in table 1, which are more COPD specific. It offers modest performance, and exceeds alternative generic scores that have undergone validation.^3^ ^9^ A generic score such as LACE is preferable to a disease-specific score, unless the latter offers superior performance.

In common with previous studies, we selected ‘death without readmission’ as a combined primary outcome with ‘readmission’. This is justified as patients who die without readmission are likely to have been readmitted had the clinical deterioration been recognised in time, and death without readmission and readmission share similar predictors.

We selected a 90-day timeframe for our primary outcome as this covers the high-risk period. In patients hospitalised due to AECOPD who survive to discharge, one-third are readmitted within 90 days^2^ and the risk of further exacerbation and readmission over the 8–12 weeks postdischarge outweighs the risk over the subsequent year.^10^ ^11^ Such events are associated with substantial risk of death, adverse qualify of life^12–15^ and high healthcare costs.^16^

The aims of the present study were:

1. To develop and perform internal and external validation of a tool to predict 90-day readmission or death without readmission in patients discharged from hospital following admission with AECOPD.
2. To assess tool performance at 30 days, and compare the new tool with other prognostic scores (table 1) at 30 and 90 days.

## Methods

### Study design and participation

The study populations comprise consecutive patients admitted with AECOPD within the DECAF derivation^17^ and validation^18^ cohorts who survived to discharge. Initially, a prediction model was developed in two UK hospitals (the derivation cohort); the performance of this model was then assessed in: (a) the same hospitals, but over a later time frame (‘internal validation cohort’; temporal validation) and (b) four further UK hospitals (‘external validation cohort’; temporal and geographical validation). External sites were chosen to ensure variation in COPD prevalence, socioeconomic factors, rurality and structure of care. This readmission study was a prespecified aim of this programme of research.^17^ ^18^

Patients in the derivation and external validation cohorts were recruited prospectively, with review of coding records to maximise capture. In the internal validation cohort, patients were identified retrospectively from a broad coding records search and review of COPD assessments routinely completed by the specialist nursing team. Patients in the derivation study were not excluded. When collecting candidate indices researchers were unaware of outcome. The internal and external validation cohorts were individually powered.

Inclusion criteria were: a primary diagnosis of an exacerbation of COPD; spirometric evidence of airflow obstruction; age 35 years or older and smoking history of 10 or more cigarette pack-years. Exclusion criteria were: previous inclusion in the same cohort, and any illness (other than COPD) likely to limit survival to <1 year.

### Data collection

Most sociodemographic and clinical data were collected at the time of admission; additional indices collected up to the point of discharge included length of stay and treatment with non-invasive ventilation. Candidate predictors included stable demographic, clinical and functional indices, including markers of frailty. The 90-day readmission and mortality data were collected from medical records. The selection of potential predictors of readmission/death without readmission was informed by a systematic literature search and clinical plausibility.^19^ Those predictors that are not routinely assessed or available at admission were appropriately excluded to avoid bias from missing data and to ensure both generalisability and ease of application of the final tool.^20^ ^21^ When two or more indices were closely related, the most appropriate was selected (collinearity—see the Statistical methods section). Final candidate indices assessed are shown in table 2.

**Table 2:** Demographics and candidate predictors by cohort

	Derivation	Internal validation	External validation	p Value
Number of patients, n	824	802	791	N/A
Sociodemographic details				
Female, %	54.2	56.4	51.5	0.14
Age*	72.3 (9.9)	73.1 (10.2)	72.2 (10.4)	0.14
Institutional care, %	5.2	6.0	3.0	0.013
Cigarette pack-years, n†	45 (32–60)	40 (30–56)	40 (30–60)	<0.001
Preadmission details				
eMRCD†	4 (3–5a)	5a (4–5a)	5a (4–5a)	<0.001
One or more admissions previous year, %	48.2	40.5	56.6	<0.001
Weight loss >5%, %	21.6	11.9	18.5	<0.001
FEV_1_ %predicted*	44.5 (18.1)	48.4 (19.2)	43.0 (16.9)	<0.001
Long-term oxygen, %	11.3	15.6	17.3	0.002
Long-term prednisolone, %	8.7	7.4	7.8	0.58
Left ventricular failure, %	7.4	10.7	12.3	0.003
Cor pulmonale, %	9.8	6.1	8.0	0.022
Diabetes, %	14.7	11.6	14.3	0.13
Chronic kidney disease, %	5.7	11.3	13.4	<0.001
Cerebrovascular disease, %	12.6	12.7	11.0	0.52
Atrial fibrillation, %	10.9	16.0	15.8	0.003
Asthma, %	5.1	7.2	10.2	<0.001
Cognitive impairment, %	4.6	4.4	5.4	0.58
Admission details				
Length of stay, n†	6 (4–11)	5 (3–10)	4 (2–8)	<0.001
Radiographic consolidation, %	29.9	29.7	23.0	0.004
Ineffective cough, %	9.3	9.6	3.4	<0.001
pH <7.35, %	20.3	15.0	14.5	0.14
Non-invasive ventilation treatment, %	17.8	13.7	12.6	0.011

*Mean (SD).

†Median (IQR).

p Value compares proportions, means and median values across all three groups.

eMRCD, extended MRC dyspnoea score.

In prognostic research, it is important that both the definitions of indices and methods of measurement are clear.^21^ All sites received data collection guides, which included information on sources of data, the definitions of diseases and terms and time-points for collection. We assessed stable state breathlessness using the extended MRC dyspnoea score (eMRCD). This includes a measure of frailty (ability to wash and dress independently).^18^ eMRCD assesses breathlessness ‘on a good day’ within the previous 3 months. Key differences to the traditional tool include: (a) use of the term ‘unable to leave the house unassisted’ as opposed to ‘house bound’; (b) division of the most severe category into eMRCD 5a and 5b patients, depending on whether the person requires help with both washing and dressing and (c) clear transition between levels.

Left ventricular failure (LVF) referred to a preadmission diagnosis based on echocardiograph as per the European Society of Cardiology guidelines.^22^ Three patients had LVF confirmed by a different modality (CT cardiac angiography n=1, cardiac MRI n=2).

For cor pulmonale, a clinical diagnosis was accepted, in the absence of an echocardiograph. First, this allowed for a new diagnosis of cor pulmonale at admission based on clinical assessment alone, which is consistent with usual clinical practice. Second, echocardiograph alone lacks precision to diagnose cor pulmonale in comparison with heart catheterisation, which is the gold standard.^23^ There were 193 patients who had a diagnosis of cor pulmonale, of whom 62 had preadmission echocardiographs showing cor pulmonale. Cor pulmonale on echocardiograph was defined as right ventricular impairment or a raised pulmonary artery systolic pressure of 35 mm Hg or more in association with lung disease.

Admission and readmission were defined as an admission to a hospital ward outwith the emergency department. Clinical care was not influenced by the research team, and no additional tests were performed.

### Statistical methods

The derivation cohort was adequately powered based on the minimum expected events per index (events per index: recommended=10 or more; observed=14).^24^ The available populations from the DECAF internal and external validation cohorts were individually powered on mortality. Given the higher event rate for readmission/death without readmission, both the internal and external validation cohorts were robustly powered, with a sample size of 227 required for an expected sensitivity of 70%, an SE for this estimate of 5% and an event rate of 37%.^25^ Baseline population characteristics and outcome were described using proportions, means with SDs or medians with IQRs, and compared using Fisher's exact test, analysis of variance and Kruskal-Wallis test. Logistic regression models were compared with Akaike Information Criterion (AIC).

Multiple imputation was used for predictor indices with <20% missing data, using IBM SPSS statistics V.22 to create five datasets and results were pooled using Rubin's method.^26^ Data were imputed using the Markov Chain Monte Carlo method, with linear and logistic regression for continuous and categorical indices. There was no missing data for outcome. Regression analysis between predictors and missing data supported the assumption that data were missing at random.^20^ ^27^ A large number of indices (n=67) were included in the imputation model. Multicollinearity between predictor indices was addressed^28^ and indices were dichotomised or categorised by visual inspection of the receiver operating characteristic (ROC) curve, a clinically relevant cut-off, or a median split (see online supplementary figure E1).^17^ To develop the final model, logistic regression using backward elimination was performed, with a probability of 0.1 for exclusion,^29^ and scores were assigned based on their regression coefficients.^30^ As prediction is about estimation, it is reasonable to include predictors with p values >0.05, otherwise strong predictors that are rare may be inappropriately excluded.^20^ The discrimination of the model was assessed by measurement of the area under the ROC (AUROC) curve in each cohort, and compared with other clinical scores (table 1) by the method of DeLong *et al* with and without multiple imputation.^31^

10.1136/thoraxjnl-2016-209298.supp1 supplementary data

Calibration was assessed by the Hosmer-Lemeshow goodness-of-fit test,^32^ by comparing the full regression model to the weighted model, and by comparing outcomes and re-examining the score assigned to individual indices, across all three cohorts. Kaplan-Meier and the log rank test were used for time to events.

Analyses were performed using IBM SPSS statistics V.22 and SigmaPlot V.12.3.

## Results

### Missing data

Of the candidate indices shown in table 2, all had 1% or less missing data for each cohort except for pH (derivation 6.6%, internal validation 9.4% and external validation 16.2%), weight loss (derivation 2.9%, internal validation 1.4%, external validation 12.4%), admissions per year (derivation 0%, internal validation 0%, external validation 2.1%) and cough effectiveness (derivation 0%, internal validation 0.37%, external validation 1.5%). Missing data for all indices, prognostic scores and patients are shown in online supplementary table E1.

### Patient characteristics

In the derivation, internal validation and external validation cohorts, 824 (December 2008–June 2010), 802 (January 2012–May 2013) and 791 (April 2013–May 2014) patients survived to hospital discharge, of whom 309 (37.5%), 297 (37.0%) and 330 (41.7%) were readmitted or died within 90 days of discharge. The population characteristics of each cohort are shown and compared in table 2. A diverse population of patients with AECOPD were recruited, exemplified by significant differences between cohorts in median eMRCD score, admissions in the previous year and proportions with LVF and cor pulmonale. The population characteristics of individual hospitals are described elsewhere.^17^ ^18^

### Development of a predictive score

The following indices were categorised: age <80 or 80+; cigarette pack-years <45 or 45+; eMRCD score 1–3, 4, 5a or 5b; FEV_1_ %predicted <50 or 50+; previous admissions (<2 or 2+ in the past year) and length of stay as per the LACE study (0, 1, 2, 3, 4–6, 7–13, or 14+ days).^8^ All candidate indices were analysed using backwards multivariate logistic regression (table 2). Weight loss was not entered into the model due to the high rate of missing data. Indices with high missing data rates may provide biased estimates as the test may only be performed in select patients; furthermore, collecting this index was labour intensive, a problem which would likely recur in clinical practice.^29^

The indices retained in the final model were: **P**revious admissions, **e**MRCD score, **A**ge 80 or more, cor pulmonale (‘**R**ight ventricular failure’) and **L**eft ventricular failure, and were collectively named the PEARL score (table 3, and see online supplementary table E2). The PEARL regression equation was compared within the derivation cohort by AIC, entering age as a categorical and continuous variable: PEARL_age catergorical_ AIC=940.4, PEARL_age continuous_ AIC=940.8. This is the recommended approach to compare the relative quality of two related models,^21^ and shows no difference, supporting the categorisation of age for ease of application of the score.

**Table 3:** Predictors of 90-day readmission or death in the derivation cohort, the PEARL score

	Derivation cohort				All cohorts	
PEARL indices	B	p Value	OR (95% CI)	Weighting	B	Updated weighting
Previous admissions (2+)	1.04	<0.001	2.84 (1.98 to 4.07)	2	1.14	3
eMRCD score 4	0.67	0.002	1.96 (1.29 to 2.98)	1	0.37	1
eMRCD score 5a	1.13	<0.001	3.10 (1.89 to 5.10)	2	0.85	2
eMRCD score 5b	2.02	<0.001	7.51 (4.17 to 13.52)	3	1.09	3
Age 80 or more	0.38	0.032	1.47 (1.03 to 2.08)	1	0.38	1
Right ventricular failure	0.50	0.050	1.66 (1.00 to 2.74)	1	0.63	1
Left ventricular failure	0.52	0.080	1.68 (0.94 to 3.00)	1	0.52	1
Constant	−0.78	<0.001	0.46 (0.36 to 0.58)		−0.95	
Maximum PEARL score						9

Hosmer-Lemeshow statistic=0.83, Nagelkerke r^2^=0.21.

As continuous variables were dichotomised, primarily to ensure ease of use, the regression coefficients (the column entitled ‘B’ in table 3) show the relative contribution of each index. The coefficients were used to assign initial weights to each index in the derivation cohort. There are various approaches to adjust models to improve prediction and generalisability^27^ ^33^; this can involve combining derivation and validation data sets.^27^ The assigned weights were re-evaluated after pooling all three cohorts. The original weightings assigned in the derivation cohort were appropriate, except ‘previous admissions’, which should optimally be weighted as three (table 3).

### Performance and calibration of the PEARL score

The AUROC for the PEARL score for 90-day readmission/death without readmission was: derivation=0.73 (95% CI 0.70 to 0.77); internal validation=0.68 (95% CI 0.64 to 0.72) and external validation=0.70 (95% CI 0.66 to 0.73). In all three cohorts combined, the AUROC for 90-day readmission/death without readmission was 0.70 (95% CI 0.68 to 0.73). For 90-day readmission, only (not including death) the AUROC was 0.69 (95% CI 0.67 to 0.71).

The risk of readmission or postdischarge death increases with higher PEARL scores (table 4). Further details of all cohorts, and data on readmission and death as lone outcome are shown in the online supplementary table E3; across all three cohorts, risk was similar with all p values >0.05 showing that predictions are consistent. We grouped scores into low-risk (0–1), intermediate-risk (2–4) and high-risk (5+) PEARL scores. Sensitivity and 1–specificity for the PEARL score are shown in the online supplementary table E4. In the low-risk group (PEARL 0–1), only 2.5% (22/890) died postdischarge within 90 days.

**Table 4:** Ninety-day death or readmission probability by PEARL score

Risk	PEARL score	Derivation cohort, % (n)	Validation cohort, % (n)	All cohorts, % by risk group
Low	0	15.1 (25/166)	16.4 (29/177)	20.7 (184/890)
	1	23.6 (49/208)	23.9 (81/339)	
Intermediate	2	33.8 (48/142)	36.3 (116/320)	42.1 (454/1078)
	3	51.2 (44/86)	41.7 (111/266)	
	4	59.1 (55/93)	46.8 (80/171)	
High	5	65.2 (43/66)	60.1 (95/158)	66.4 (298/449)
	6	67.5 (27/40)	69.2 (63/91)	
	7	72.2 (13/18)	70.2 (40/57)	
	8	100 (4/4)	77.8 (7/9)	
	9	100 (1/1)	100 (5/5)	
Total		37.5 (309/824)	39.4 (627/1593)	38.7 (936/2417)

**Figure 1:**
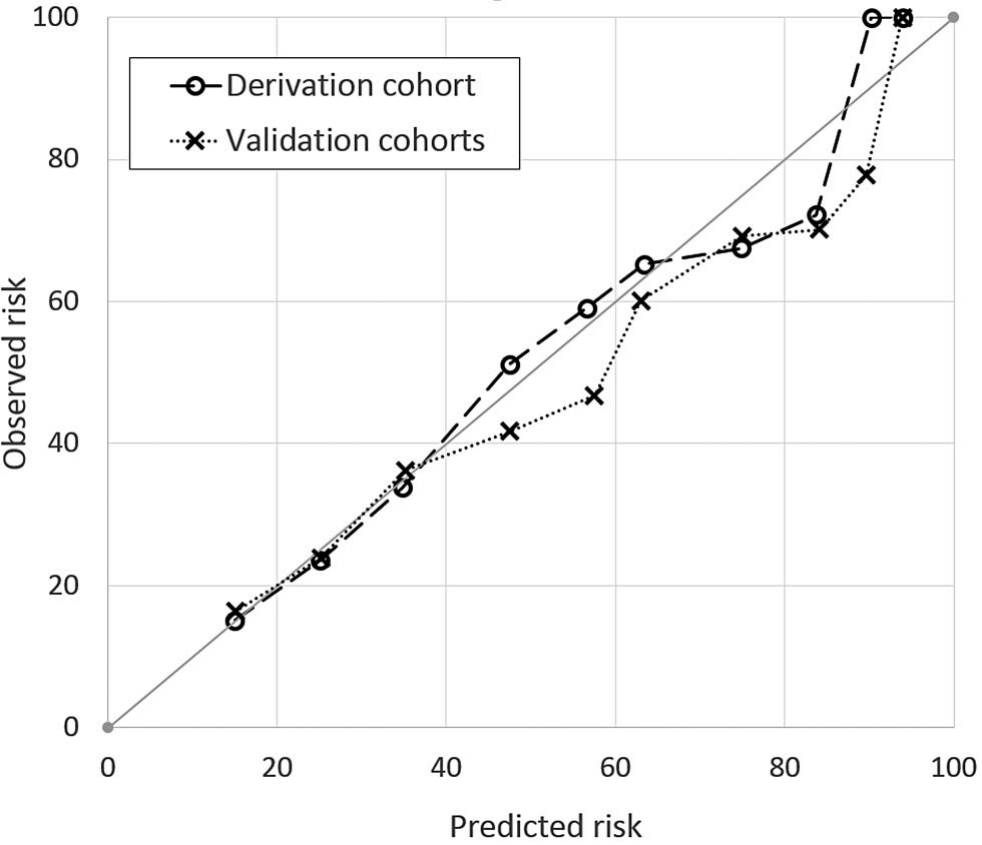
Calibration curve showing predicted risk compared with observed risk by PEARL score.

Calibration was further assessed by plotting ‘expected probability’ (calculated from the full regression equation) against the ‘observed probability’. Calibration tends to perform best in derivation cohorts, so the derivation and validation cohorts were plotted separately. Again, PEARL was well calibrated (perfect calibration would fall on the 45° line; see figure 1).

### Comparison with other prognostic scores

**Figure 2:**
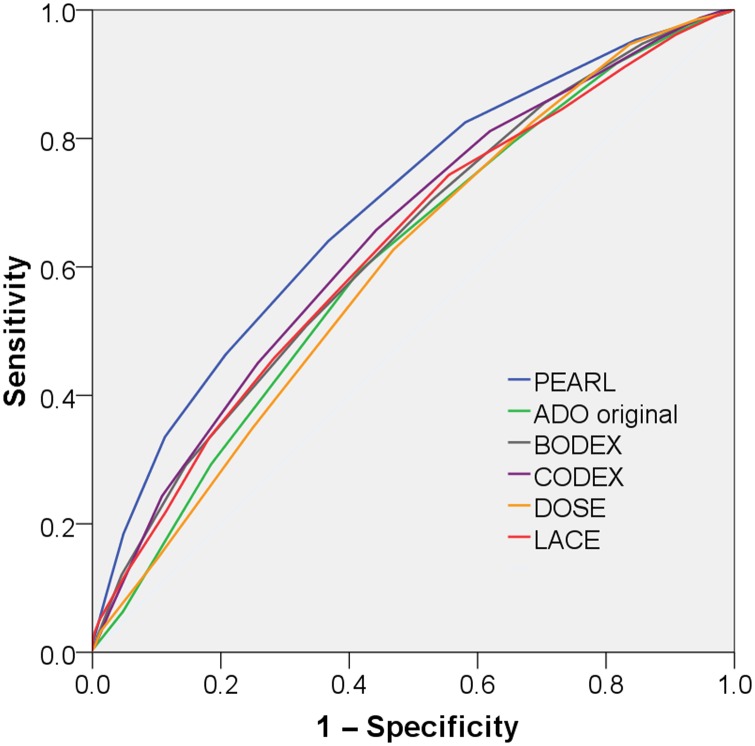
Receiver operating characteristic curves for PEARL, ADO, BODEX, CODEX, DOSE and LACE for 90-day readmission or death, validation cohorts combined.

The ROC curves for each prognostic score are shown in figure 2 for the validation cohorts combined. Comparison within a derivation cohort favours the developed tool, so the derivation cohort is not included within the graph. This shows that the ROC curve area is higher for PEARL than the other scores.

Table 5 shows the comparison between PEARL and all other tools for each individual cohort. PEARL was superior to ADO, BODEX, DOSE and LACE in all three cohorts, and to CODEX within the derivation and external validation cohorts. Results were unchanged with complete case analysis. Thirty-day comparisons are shown online (see online supplementary supplementary table E6).

**Table 5:** Ninety-day readmission or death, AUROC curves, with data imputation

Prognostic score	Derivation	Internal validation	External validation
PEARL	0.73 (0.70 to 0.77)	0.68 (0.64 to 0.72)	0.70 (0.66 to 0.73)
ADO	0.67 (0.63 to 0.71)*	0.64 (0.60 to 0.67)†	0.58 (0.54 to 0.62)*
BODEX	0.65 (0.61 to 0.69)*	0.64 (0.60 to 0.68)‡	0.62 (0.58 to 0.66)*
CODEX	0.69 (0.65 to 0.73)†	0.66 (0.63 to 0.70) NS	0.62 (0.58 to 0.66)*
DOSE	0.63 (0.59 to 0.67)*	0.59 (0.55 to 0.64)*	0.61 (0.57 to 0.65)*
LACE	0.65 (0.61 to 0.69)*	0.61 (0.57 to 0.65)†	0.65 (0.61 to 0.68)‡

AUROC curves (and 95% CIs) of each score compared with PEARL by method of DeLong *et al*: *<0.001, †<0.01, ‡<0.05.

Missing data >20% for BODEX and DOSE. On complete case analysis, BODEX=0.63 (0.59–0.67), DOSE=0.60 (0.53–0.66).

NS, not significant.

### Time to death or readmission, and readmission frequency

**Figure 3:**
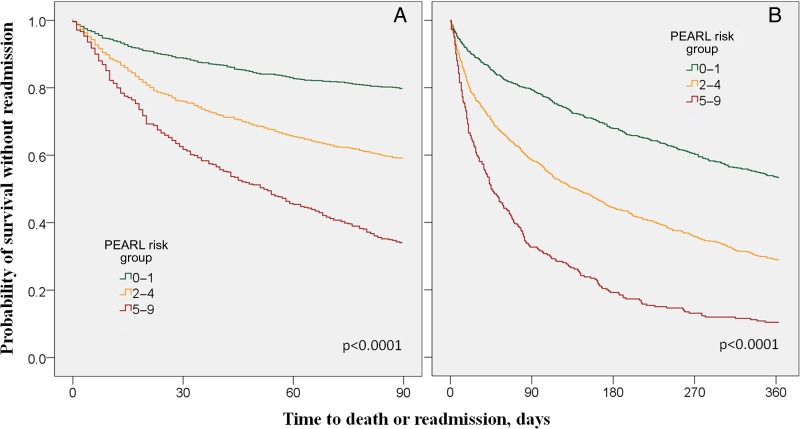
Time to readmission or death, by PEARL risk group: (A) in all cohorts up to 90 days, (B) in the derivation and internal validation cohort up to 365 days (comparison using the log rank test).

Time to death or readmission was available for 90 days in all three cohorts and for 1 year in the derivation and internal validation cohorts. Higher PEARL risk groups were associated with a shorter time to death or readmission (figure 3 and see online supplementary table E7).

PEARL risk group identifies those at risk of frequent admissions. For risk groups 0–1, 2–4 and 5–9, the median (IQR) number of readmission was 0 (0–1), 1 (0–2) and 2 (1–3). When adjusted for death (time exposed to readmission), the risk was 0 (0–1.8), 1 (0–3) and 3 (1–6), respectively.

## Discussion

We have developed and validated a model to predict 90-day readmission/death without readmission in patients hospitalised with an AECOPD, the ‘PEARL score’. The tool was designed to be easily applied at the bedside using indices routinely available at admission, and performance was superior to alternative scores. The risk of readmission/readmission without death was considerably higher in the first 90 days than during the rest of the year, both overall and within the moderate-risk and high-risk PEARL groups, which justifies our chosen timeframe. Rates of readmission were similar to those seen in the European National Audit 2016.^34^ Our composite end point is more appropriate than readmission alone, as the latter would include both those who are neither readmitted nor die and those who die without readmission in the ‘favourable’ outcome group. Accurate risk stratification of patients should help efficiently direct resources aimed to reduce readmissions, such as supported discharge services, pulmonary rehabilitation, education programmes and possibly azithromycin therapy, although the impact of these strategies requires assessment. Furthermore, identification of patients who are at risk of death without readmission may allow services to be put in place to facilitate early recognition of deterioration and readmission.

The study has a number of strengths, most importantly consecutive recruitment of patients and high case ascertainment. This is supported by excellent recruitment rates at all sites which were substantially higher than the 2015 UK national COPD audit, as detailed previously.^18^ Generalisability is supported by consistent performance in three cohorts, including the prospective external validation cohort (the ‘gold standard’ for assessing performance). The six hospitals that took part had different structures of care and varied populations, with respect to readmission avoidance schemes, COPD prevalence, socioeconomics and rurality. Furthermore, data were collected by a variety of healthcare professionals, including physicians and specialist nurses.

The eligibility criteria were inclusive; few patients were excluded due to poor prognosis (expected survival <1 year for an illness other than COPD): in the internal validation cohort, for example, this comprised only 27 patients (3.4%), principally due to metastatic malignancy. Definitions were aligned with usual clinical practice, and were pragmatic to reduce missing data and the consequent risk of bias. This approach is regarded as a key strength in prognostic research.^20^ ^21^ Primary outcome data were available in all patients, missing data rates were low and multiple imputation and complete case analyses showed that results were robust. Further study strengths can be seen in the online supplement, ‘The CHARMS checklist’,^21^ which provides a framework to critique prognostic studies.

There are a number of limitations within the study. Most patients in the internal validation cohort were identified retrospectively, which may have compromised performance. However, the risk of any consequent bias is probably low as the relevant indices were recorded during the patients' admission prior to the outcome. When extracting data, researchers were blind to outcome. Furthermore, the external validation cohort was prospective and individually powered.

Varied rates of cor pulmonale and LVF may reflect inconsistency in clinical assessment, or could represent true population differences, supporting external validity. Despite variation in rates, both were associated with readmission/death without readmission in all three cohorts. Dichotomising continuous indices, such as age, allows a score to be calculated at the bedside without the full regression equation and a computer. When this approach is adopted, concern about consequent loss of prognostic strength may be raised. However, the impact of dichotomising (or categorising) continuous variables on performance may be minimal if the relation between the index and risk of outcome is non-linear, and the prognostic threshold(s) appropriately selected.^20^ The impact of dichotomising age on the performance of the PEARL score was negligible. We did not differentiate between different causes of readmission such as respiratory or non-respiratory. This would require the derivation and validation of separate scores, and based on data from our validation cohorts most admissions are respiratory (more than three in four). Furthermore, differentiating between cardiac and respiratory causes of readmission can be challenging. While PEARL performed well in all cohorts, and participating units were chosen to ensure variation in population and structure of care, confirmation of performance in healthcare settings outside the UK is desirable. A final limitation, which is common to all prognostic research, is that the strength of the association of the predictor with the outcome may vary between patients.^35^

Other prognostic research shows that accurate prediction of readmission is challenging.^9^ The discrimination of PEARL (external validation AUROC=0.70) is superior to all other tools that predict readmission or death in AECOPD, and substantially stronger than clinical judgement (AUROC=0.56–0.59).^3^ An extensive literature search was performed to ensure the inclusion of all potential predictor variables that could be easily collected at the bedside. All of the indices in the PEARL score have been previously shown to predict our outcome, except for eMRCD score. This is important as eMRCD (along with previous admissions) was the strongest predictor. In our study, associated pneumonia, non-invasive ventilation and institutional care (nursing or residential home) did not appear in the final model. This does not mean that they are not predictors, but rather that they did not add prognostic power to the PEARL indices, which were stronger predictors. Furthermore, eMRCD includes a measure of frailty which may, at least in part, capture the risk associated with such indices. The strongest predictors of readmission/death without readmission tended to be measures of underlying disease severity, frailty and comorbidity rather than measures related to the acute event. For instance, DECAF contains eosinopoenia (a marker of acute inflammation/sepsis), consolidation and acidaemia; it is an excellent predictor of acute mortality, but not medium-term and long-term outcomes in those who survive to discharge.

The lack of novel predictors, such as cardiac biomarkers, neural respiratory drive,^36^ 4 m gait speed^37^ and quadriceps size by ultrasound^38^ may be seen as a limitation. The inclusion of too many indices in model development risks overfitting and loss of performance in the validation cohorts.^20^ Furthermore, the inclusion of indices that are not routinely collected may introduce bias as missing data are large,^39^ ^40^ and any association may be due to case selection only. For example, in our study only 17% of patients had troponin tests performed, and levels were not related to outcome on univariate analysis. We were unable to capture psychological well-being, social support networks and treatment concordance. These variables are complex to measure and may reduce the usability of a tool.

Previous studies have shown a relationship between anxiety and depression and readmission,^41^ ^42^ although this was not seen in our derivation cohort (based on a preadmission clinical diagnosis). It is possible that an alternative approach to assessing anxiety, such as measurement of the Hospital Anxiety and Depression score at the point of discharge, may add predictive information.

FEV_1_ is associated with exacerbations and hospital admissions in patients with stable COPD and guides treatment,^16^ ^43^ ^44^ but in our derivation cohort it was not an independent predictor of the primary outcome. The better performance of FEV_1_ in the derivation cohorts of tools such as ADO, BODEX, CODEX and DOSE probably reflects differences in population and measured outcome.

PEARL was superior to ADO, updated ADO, BODEX, CODEX, DOSE and LACE. The CODEX study was developed in a large number of hospitals, the population is clearly described and model performance is appropriately assessed. In its derivation study, it was superior to ADO and BODEX for 90-day readmission or death, although this comparison favours CODEX.^4^ In our study, it was the second best performing tool. LACE was developed in unselected patients, rather than those with AECOPD,^9^ for 30-day (and not 90-day) outcome. Of importance, PEARL was superior at both time-points. The LACE score was selected for comparison as it was derived in a well-conducted study, demonstrated better discrimination than other generic tools and is used in some hospitals. The requirement to score the full Charlson Comorbidity Index limits the bedside application of both CODEX and LACE.

A number of studies have shown positive outcomes from interventions aimed to reduce readmission.^45–47^ There is room for further research to improve outcome, particularly in COPD.^48^ ^49^ The lowest risk group (PEARL 0–1) comprise almost a third of the population. Such risk stratification can inform research by excluding low-risk groups (of importance, the risk of death alone was only 2.5% in the low risk, PEARL 0–1 group), or by using randomisation techniques that include stratification or minimisation by risk group.

In current practice, clinician judgement is used to identify and target resources towards patients with a high readmission risk, although this judgement is known to be poor.^3^ The PEARL score offers robust and consistent prediction of 90-day readmission or death, and is superior to alternative tools. PEARL may aid clinical decision-making and resource allocation, although quantification of the impact of PEARL in terms of cost and patient outcomes requires further research.

## References

[1] Healthcare Commission. Clearing the air. A national study of chronic obstructive pulmonary disease. Commission for Healthcare Audit and Inspection, 2006.

[2] Roberts CM, Lowe D, Bucknall CE, et al Clinical audit indicators of outcome following admission to hospital with acute exacerbation of chronic obstructive pulmonary disease. Thorax 2002;57:137–41. 10.1136/thorax.57.2.137 11828043 PMC1746248

[3] Allaudeen N, Schnipper JL, Orav EJ, et al Inability of providers to predict unplanned readmissions. J Gen Intern Med 2011;26:771–6. 10.1007/s11606-011-1663-3 21399994 PMC3138589

[4] Almagro P, Soriano JB, Cabrera FJ, et al Short- and medium-term prognosis in patients hospitalized for COPD exacerbation: the CODEX index. Chest 2014;145:972–80. 10.1378/chest.13-1328 24077342

[5] Puhan MA, Garcia-Aymerich J, Frey M, et al Expansion of the prognostic assessment of patients with chronic obstructive pulmonary disease: the updated BODE index and the ADO index. Lancet 2009;374:704–11. 10.1016/S0140-6736(09)61301-5 19716962

[6] Soler-Cataluña JJ, Martínez-García MA, Sánchez LS, et al Severe exacerbations and BODE index: two independent risk factors for death in male COPD patients. Respir Med 2009;103:692–9. 10.1016/j.rmed.2008.12.005 19131231

[7] Jones RC, Donaldson GC, Chavannes NH, et al Derivation and validation of a composite index of severity in chronic obstructive pulmonary disease: the DOSE Index. Am J Respir Crit Care Med 2009;180:1189–95. 10.1164/rccm.200902-0271OC 19797160

[8] van Walraven C, Dhalla IA, Bell C, et al Derivation and validation of an index to predict early death or unplanned readmission after discharge from hospital to the community. CMAJ 2010;182:551–7. 10.1503/cmaj.091117 20194559 PMC2845681

[9] Kansagara D, Englander H, Salanitro A, et al Risk prediction models for hospital readmission: a systematic review. JAMA 2011;306:1688–98. 10.1001/jama.2011.1515 22009101 PMC3603349

[10] Hurst JR, Donaldson GC, Quint JK, et al Temporal clustering of exacerbations in chronic obstructive pulmonary disease. Am J Respir Crit Care Med 2009;179:369–74. 10.1164/rccm.200807-1067OC 19074596

[11] Suissa S, Dell'Aniello S, Ernst P Long-term natural history of chronic obstructive pulmonary disease: severe exacerbations and mortality. Thorax 2012;67:957–63. 10.1136/thoraxjnl-2011-201518 22684094 PMC3505864

[12] Seemungal TA, Donaldson GC, Paul EA, et al Effect of exacerbation on quality of life in patients with chronic obstructive pulmonary disease. Am J Respir Crit Care Med 1998;157(Pt 1):1418–22. 10.1164/ajrccm.157.5.9709032 9603117

[13] Doll H, Miravitlles M Health-related QOL in acute exacerbations of chronic bronchitis and chronic obstructive pulmonary disease: a review of the literature. Pharmacoeconomics 2005;23:345–63. 10.2165/00019053-200523040-00005 15853435

[14] Bourbeau J, Ford G, Zackon H, et al Impact on patients’ health status following early identification of a COPD exacerbation. Eur Respir J 2007;30:907–13. 10.1183/09031936.00166606 17715163

[15] Soler-Cataluña JJ, Martínez-García MA, Roman Sánchez P, et al Severe acute exacerbations and mortality in patients with chronic obstructive pulmonary disease. Thorax 2005;60:925–31. 10.1136/thx.2005.040527 16055622 PMC1747235

[16] Vestbo J, Hurd SS, Agustí AG, et al Global strategy for the diagnosis, management, and prevention of chronic obstructive pulmonary disease: GOLD executive summary. Am J Respir Crit Care Med 2013;187:347–65. 10.1164/rccm.201204-0596PP 22878278

[17] Steer J, Gibson J, Bourke SC The DECAF Score: predicting hospital mortality in exacerbations of chronic obstructive pulmonary disease. Thorax 2012;67:970–6. 10.1136/thoraxjnl-2012-202103 22895999

[18] Echevarria C, Steer J, Heslop-Marshall K, et al Validation of the DECAF score to predict hospital mortality in acute exacerbations of COPD. Thorax 2016;71:133–40. 10.1136/thoraxjnl-2015-207775 26769015 PMC4752621

[19] Steer J, Gibson GJ, Bourke SC Predicting outcomes following hospitalization for acute exacerbations of COPD. QJM 2010;103:817–29. 10.1093/qjmed/hcq126 20660633

[20] Steyerberg E Clinical prediction models, a practical approach to development, validation and updating. Springer, 2009.

[21] Moons KG, de Groot JA, Bouwmeester W, et al Critical appraisal and data extraction for systematic reviews of prediction modelling studies: The CHARMS checklist. PLoS Med 2014;11:e1001744 10.1371/journal.pmed.1001744 25314315 PMC4196729

[22] Ponikowski P, Voors AA, Anker SD, et al 2016 ESC Guidelines for the diagnosis and treatment of acute and chronic heart failure: the Task Force for the diagnosis and treatment of acute and chronic heart failure of the European Society of Cardiology (ESC). Developed with the special contribution of the Heart Failure Association (HFA) of the ESC. Eur J Heart Fail 2016;18:891–975. 10.1002/ejhf.592 27207191

[23] D'Alto M, Romeo E, Argiento P, et al Accuracy and precision of echocardiography versus right heart catheterization for the assessment of pulmonary hypertension. Int J Cardiol 2013;168:4058–62. 10.1016/j.ijcard.2013.07.005 23890907

[24] Harrell FE Jr, Lee KL, Califf RM, et al Regression modelling strategies for improved prognostic prediction. Stat Med 1984;3:143–52. 10.1002/sim.4780030207 6463451

[25] Carley S, Dosman S, Jones SR, et al Simple nomograms to calculate sample size in diagnostic studies. Emerg Med J 2005;22:180–1. 10.1136/emj.2003.011148 15735264 PMC1726700

[26] Rubin DB Multiple imputation after 18+ years. J Am Stat Assoc 1996;91:473–89. 10.1080/01621459.1996.10476908

[27] Moons KG, Altman DG, Reitsma JB, et al Transparent Reporting of a multivariable prediction model for Individual Prognosis or Diagnosis (TRIPOD): explanation and elaboration. Ann Intern Med 2015;162:W1–73. 10.7326/M14-0698 25560730

[28] Field A Discovering statistics using SPSS. 3rd edn London: SAGE, 2009.

[29] Royston P, Moons KG, Altman DG, et al Prognosis and prognostic research: developing a prognostic model. BMJ 2009;338:b604 10.1136/bmj.b604 19336487

[30] Sullivan LM, Massaro JM, D'Agostino RB Sr Presentation of multivariate data for clinical use: The Framingham Study risk score functions. Stat Med 2004;23:1631–60. 10.1002/sim.1742 15122742

[31] DeLong ER, DeLong DM, Clarke-Pearson DL Comparing the areas under two or more correlated receiver operating characteristic curves: a nonparametric approach. Biometrics 1988;44:837–45. 10.2307/2531595 3203132

[32] Hosmer DW Jr, Lemesbow S Goodness of fit tests for the multiple logistic regression model. Commun Stat 1980;9:1043–69. 10.1080/03610928008827941

[33] Steyerberg EW, Borsboom GJ, van Houwelingen HC, et al Validation and updating of predictive logistic regression models: a study on sample size and shrinkage. Stat Med 2004;23:2567–86. 10.1002/sim.1844 15287085

[34] Hartl S, Lopez-Campos JL, Pozo-Rodriguez F, et al Risk of death and readmission of hospital-admitted COPD exacerbations: European COPD Audit. Eur Respir J 2016;47:113–21. 10.1183/13993003.01391-2014 26493806

[35] Ware JH The limitations of risk factors as prognostic tools. N Engl J Med 2006;355:2615–7. 10.1056/NEJMp068249 17182986

[36] Suh ES, Mandal S, Harding R, et al Neural respiratory drive predicts clinical deterioration and safe discharge in exacerbations of COPD. Thorax 2015;70:1123–30. 10.1136/thoraxjnl-2015-207188 26194996 PMC4680187

[37] Kon SS, Jones SE, Schofield SJ, et al Gait speed and readmission following hospitalisation for acute exacerbations of COPD: a prospective study. Thorax 2015;70:1131–7. 10.1136/thoraxjnl-2015-207046 26283709

[38] Greening NJ, Harvey-Dunstan TC, Chaplin EJ, et al Bedside assessment of quadriceps muscle by ultrasound after admission for acute exacerbations of chronic respiratory disease. Am J Respir Crit Care Med 2015;192:810–16. 10.1164/rccm.201503-0535OC 26068143 PMC4613897

[39] Martins CS, Rodrigues MJO, Miranda VP, et al Prognostic value of cardiac troponin I in patients with COPD acute exacerbation. Neth J Med 2009;67:341–9. 19915228

[40] Brekke PH, Omland T, Holmedal SH, et al Troponin T elevation and long-term mortality after chronic obstructive pulmonary disease exacerbation. Eur Respir J 2008;31:563–70. 10.1183/09031936.00015807 18032444

[41] Laurin C, Moullec G, Bacon SL, et al Impact of anxiety and depression on chronic obstructive pulmonary disease exacerbation risk. Am J Respir Crit Care Med 2012;185:918–23. 10.1164/rccm.201105-0939PP 22246177

[42] Pooler A, Beech R Examining the relationship between anxiety and depression and exacerbations of COPD which result in hospital admission: a systematic review. Int J Chron Obstruct Pulmon Dis 2014;9:315–30. 10.2147/COPD.S53255 24729698 PMC3974694

[43] Agusti A, Edwards LD, Celli B, et al Characteristics, stability and outcomes of the 2011 GOLD COPD groups in the ECLIPSE cohort. Eur Respir J 2013;42:636–46. 10.1183/09031936.00195212 23766334

[44] Calverley PM, Anderson JA, Celli B, et al Salmeterol and fluticasone propionate and survival in chronic obstructive pulmonary disease. N Engl J Med 2007;356:775–89. 10.1056/NEJMoa063070 17314337

[45] Naylor M, Brooten D, Jones R, et al Comprehensive discharge planning for the hospitalized elderly. A randomized clinical trial. Ann Intern Med 1994;120:999–1006. 8185149 10.7326/0003-4819-120-12-199406150-00005

[46] Coleman EA, Parry C, Chalmers S, et al The care transitions intervention: results of a randomized controlled trial. Arch Intern Med 2006;166:1822–8. 10.1001/archinte.166.17.1822 17000937

[47] Rich MW, Beckham V, Wittenberg C, et al A multidisciplinary intervention to prevent the readmission of elderly patients with congestive heart failure. N Engl J Med 1995;333:1190–5. 10.1056/NEJM199511023331806 7565975

[48] Bucknall CE, Miller G, Lloyd SM, et al Glasgow supported self-management trial (GSuST) for patients with moderate to severe COPD: randomised controlled trial. BMJ 2012;344:e1060 10.1136/bmj.e1060 22395923 PMC3295724

[49] Fan VS, Gaziano JM, Lew R, et al A comprehensive care management program to prevent chronic obstructive pulmonary disease hospitalizations: a randomized, controlled trial. Ann Intern Med 2012;156:673–83. 10.7326/0003-4819-156-10-201205150-00003 22586006

