# Supplementary material for: The PEARL score predicts 90-day readmission or death after hospitalisation for acute exacerbation of COPD
Source: Thorax. 2017 Feb 24;72(8):686–93. doi: 10.1136/thoraxjnl-2016-209298 (PMC5537524; doi:10.1136/thoraxjnl-2016-209298)
Supplement: supplementary data [file thoraxjnl-2016-209298supp001.pdf]

ONLINE SUPPLEMENT

Table E1. Percentage of missing data per variable in each cohort

	Missing data, %		
	Derivation	Internal validation	External validation
Number or patients, n	824	802	791
Patients with missing data	2.9	3.6	16.9
Excluding weight loss	0	2.2	6.4
Socio-demographic details			
Gender	0	0	0
Age	0	0	0
Residence	0	0	0
Cigarette pack years	0	0	0
Preadmission details			
eMRCD	0	0	0
Admissions in previous year	0	0	2.1
Recent weight loss	2.9	1.4	12.4
FEV1 % predicted	0	0.75	1.5
Long term oxygen	0	0	0.38
Long term prednisolone	0	0	0.63
Left ventricular failure	0	0	0.25
Cor pulmonale	0	0	0.25
Diabetes	0	0	0.38
Chronic Kidney Disease	0	0	0.13
Cerebrovascular Disease	0	0	0.51
Atrial Fibrillation	0	0	0
Asthma	0	0	0.25
Cognitive impairment	0	0	0.38
Admission details			
Admission hospital	0	0	0
Length of stay	0	0	0.76
Radiographic consolidation	0	0	0
Cough effectiveness	0	0.37	1.5
pH	6.6	9.4	16.2
NIV	0	0	1.0
Variables from other prognostic with missing data			
BMI	2.9	21.3	25.4
Exacerbations	11.9	61.5	18.2
Severe exacerbations	0	0	1.3
Current smoking status	0	0.62	0.38
Missing data by prognostic score			
PEARL	0	0	2.4
ADO (original and updated)	0	1.9	1.5
BODEX	2.9	22.1	26.2
CODEX	0	1.9	2.7
DOSE	11.9	62.6	19.1
LACE	0	0	1.9

Table E2. The PEARL score

Index	Score
Previous admissions (2 or more within past year)	0 / 3
Dyspnoea	
eMRCD 1-3	0
eMRCD 4	1
eMRCD 5a	2
eMRCD 5b	3
Age 80 or more	0 / 1
Right ventricular failure (Cor pulmonale)	0 / 1
Left ventricular failure	0 / 1
Total PEARL score	9

Table E3. Online supplemental table showing observed probabilities for all cohorts, and outcome at 30 and 90 days.

“Expected probability” refers to the predicted risk of 90-day readmission or death calculated from the full regression model, and the “observed probability” is the measured outcome rate for each PEARL score in the each cohort.

Risk	Score		Expected probability derivation cohort	Observed probability derivation cohort	Observed probability internal validation cohort	Observed probability external validation cohort	P value	Readmission or death within 90 days	Death alone within 90 days	Readmission alone within 90 days	Readmission or death within 30 days	Total (all patients)
Low	**0**	n		25 / 166	17 / 97	12 / 80	0.84	54	7	50	32	343
		%	15.0	15.1	17.5	15.0		**15.7**	2.0	14.6	9.3	
	**1**	n		49 / 208	47 / 193	34 / 146	0.98	130	15	125	69	547
		%	25.1	23.6	24.4	23.3		**23.8**	2.7	22.9	12.6	
Intermediate	**2**	n		48 / 142	60 / 178	56 / 142	0.51	164	46	149	93	462
		%	34.8	33.8	33.7	39.4		**35.5**	10.0	32.3	20.1	
	**3**	n		44 / 86	51 / 126	60 / 140	0.29	155	58	125	100	352
		%	47.5	51.2	40.5	42.9		**44.0**	16.5	35.5	28.4	
	**4**	n		55 / 93	35 / 77	45 / 94	0.16	135	33	120	69	264
		%	56.6	59.1	45.5	47.9		**51.1**	12.5	45.5	26.1	
High risk	**5**	n		43 / 66	40 / 63	55 / 95	0.62	138	24	127	79	224
		%	63.4	65.2	63.5	57.9		**61.6**	10.7	56.7	35.3	
	**6**	n		27 / 40	28 / 40	35 / 51	1.00	90	28	83	51	131
		%	74.8	67.5	70.0	68.6		**68.7**	21.4	63.4	38.9	
	**7**	n		13 / 18	16 / 24	24 / 33	0.90	53	14	49	31	75
		%	83.7	72.2	66.7	72.7		**70.7**	18.7	65.3	41.3	
	**8**	n		4 / 4	1 / 2	6 / 7	0.37	11	6	10	6	13
		%	90.2	100	50	85.7		**84.6**	46.2	76.9	46.2	
	**9**	n		1 / 1	2 / 2	3 / 3	N/A	6	3	6	5	6
		%	93.9	100	100	100		**100**	50	100	83.3	
Total				309 / 824	297 / 802	330 / 791	0.11	936	234	844	535	2417
%			37.5	37.5	37.0	41.7		**38.7**	9.7	34.9	22.1	

P value compares the three observed proportions by Fishers test (the comparison of expected to observed probabilities is shown separately by the Hosmer-Lemeshow statistic and the calibration curve).

Table E4. Sensitivity and 1-specificty for the PEARL score, derivation cohort.

PEARL score	Sensitivity	1 – Specificity
0	1.00	1.00
1	0.92	0.73
2	0.76	0.42
3	0.61	0.24
4	0.46	0.15
5	0.29	0.080
6	0.15	0.035
7	0.058	0.010
8	0.016	<0.001
9	0.003	<0.001

Table E5. 30-day readmission or death, AUROC curves, with data imputation

Prognostic score	Derivation	Comparison to PEARL, p value	Internal validation	Comparison to PEARL, p value	External validation	Comparison to PEARL, p value
PEARL	0.70 (0.66-0.74)	N/A	0.64 (0.60-0.69)	N/A	0.64 (0.60-0.69)	N/A
ADO	0.63 (0.59-0.68)	0.002	0.61 (0.56-0.65)	0.082	0.56 (0.51-0.60)	<0.001
BODEX	0.63 (0.59-0.68)	0.003	0.61 (0.57-0.66)	0.16	0.58 (0.53-0.62)	0.005
CODEX	0.65 (0.61-0.70)	0.014	0.62 (0.58-0.67)	0.28	0.59 (0.54-0.63)	0.006
DOSE	0.61 (0.57-0.66)	<0.001	0.58 (0.53-0.63)	0.016	0.59 (0.54-0.63)	0.021
LACE	0.66 (0.61-0.70)	0.087	0.61 (0.57-0.66)	0.26	0.59 (0.55-0.64)	0.038

AUROC curves (and 95% confidence intervals) of each prognostic score compared to DECAF by method of DeLong.

Missing data >20% for BODEX and DOSE. On complete case analysis, BODEX= 0.57 (0.52-0.62), DOSE= 0.61 (0.53-0.68)

Table E6. 30-day readmission or death, AUROC curves, for updated ADO score, eMRCD and DECAF

	Prognostic score	Derivation	Internal validation	External validation
30 day readmission or death	PEARL score	0.70 (0.66-0.74)	0.64 (0.60-0.69)	0.64 (0.60-0.69)
	ADO (updated)	0.62 (0.57-0.67)	0.60 (0.55-0.64)	0.56 (0.51-0.60)
	eMRCD score	0.64 (0.59-0.68)	0.60 (0.55-0.64)	0.57 (0.53-0.62)
	DECAF score	0.57 (0.53-0.62)	0.59 (0.54-0.64)	0.54 (0.49-0.59)
90 day readmission or death	PEARL score	0.73 (0.70-0.77)	0.68 (0.64-0.72)	0.70 (0.66-0.73)
	ADO (updated)	0.65 (0.61-0.69)	0.63 (0.59-0.66)	0.58 (0.54-0.62)
	eMRCD score	0.68 (0.65-0.72)	0.61 (0.57-0.65)	0.61 (0.57-0.65)
	DECAF score	0.59 (0.55-0.63)	0.57 (0.53-0.61)	0.57 (0.53-0.61)
PEARL AUROC (95% CI) are superior to all the above scores ($p<0.05$) by method of DeLong.				

Table E7. Time to readmission or death, by PEARL risk group, derivation and internal validation cohort

PEARL risk group	PEARL score	Days to readmission or death, % 0 to 30	31 to 60	61 to 90	91 to 365	No readmission or death, %	Total, n
Low	0-1	11.3%	6.5%	3.0%	25.9%	53.3%	664
Intermediate	2-4	24.1%	10.0%	7.7%	29.2%	29.1%	702
High	5-9	40.4%	15.4%	11.5%	22.3%	10.4%	250
	Total	21.5%	9.4%	6.4%	26.8%	36.0%	1626

Figure E1. ROC curves for eMRCD score, admissions in previous year, and age as continuous indices with arrows showing selected cut-off.

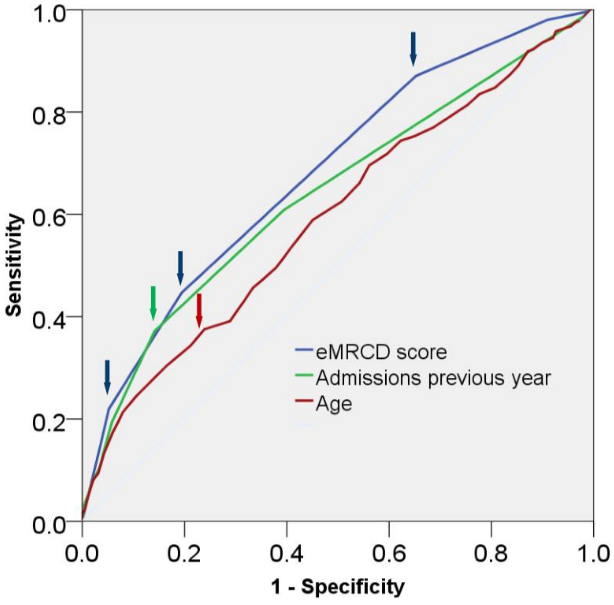

CHARMS CHECKLIST

Source of Data

1) Source of data (e.g. cohort, case-control, randomised trial participants, or registry data)

- The derivation and external validation cohorts were prospective. The internal validation cohort was retrospective.

Participants

1) Participant eligibility and recruitment method (consecutive participants, location, number of centres, setting, inclusion and exclusion criteria)

Eligible patients analysis

- Patients who did not survive to discharge were appropriately excluded as the main outcome was readmission/ death without readmission at 90 days from discharge. Otherwise, all patients were included in the derivation cohort. The primary outcome for the three cohorts was for inpatient mortality, which led to the development of the DECAF score. The readmission analysis was a pre-specified study. In the validation cohort, those that did have complete data for all DECAF indices were not included in the analysis, although this was only 1% of the population, and mainly comprised of patients that had oxygen saturations sufficiently low to warrant arterial blood gas analysis but that declined this investigation.

Eligible patients excluded

- Exclusion criteria were few. For the internal validation cohort, patients were not eligible as follows: survival <1 year n=27 (twelve lung cancer, three end stage dementia, three metastatic cancer, two metastatic bladder cancer, two idiopathic pulmonary fibrosis, one metastatic renal cancer, one metastatic bower cancer, one metastatic rectal cancer, one oesophageal cancer, and one mesenteric cancer patient), less than ten pack year smoking history n=24, spirometry not obstructive= 42. Ten patients had no ABG results, but had supplemental oxygen or oxygen saturations that were too low to assume a DECAF acidaemia score of zero. One patient had no eosinophil count. Robust data for the derivation and external validation cohort is unavailable.

Consecutive patients

- Extensive efforts were made to capture consecutive patients, including a broad coding search. Patients were captured by daily screening (Monday to Friday) on admission units and medical wards (derivation and external validation cohorts) by a dedicated team. In the internal validation cohort, patients were mainly identified retrospectively using a broad coding search, with cross referencing to clinical staff whose role it is to review patients with exacerbation of COPD. In the internal validation cohort, a dedicated team screened the admission units and medical wards for three months and compared patient capture to the coding records search and clinical team capture. Only one patient was identified by daily screening that was missed by coding or the clinical team.

Location, centres, setting, and inclusion and exclusion criteria

- Six UK centres were involved: the same two sites that were included in the derivation cohort took part in the internal validation cohort, and four geographical distinct hospitals took part in the external validation cohort. All patients in the study were recruited from secondary care. Inclusion and exclusion criteria are described.

2) Participant description

- Detailed description of participants by different sites in DECAF validation study.[18] A detailed description of patients in each cohort is shown in table 2.

3) Details of treatments received, if relevant

- Treatments to reduce hospital readmission include smoking cessation, inhaled corticosteroids, long-acting beta agonists, and long-acting muscarinic agonists, and pulmonary rehabilitation. The score is intended to be used to inform management, so including many acute treatments as predictors is not appropriate. Long term oxygen and long term prednisolone were included in model development. NIV treatment is based on fairly objective criteria based on pH, and has been previously shown to be predictive, so was included (see table 2). The research team did not influence clinical treatment.

4) Study dates

- Dates for recruitment period of each hospital discussed in previous publications.[17 18]

Outcome to be predicted

1) Definition and method for measurement of outcome

- Readmission/ death without readmission 90 days from discharge in patients surviving to discharge. Readmission was clearly defined- a patient had to be admitted to hospital and reviewed by a member of the clinical team.

2) Was the same outcome definition (and method for measurement) in all patients?
- Yes.

3) Type of outcome single or combined endpoints?
- Combined outcome. We did not wish to create a score that identified those at risk of readmission, but missed those at risk of death without readmission, as some of these deaths may be preventable. Predictors of readmission and death are similar.

4) Was the outcome assessed without knowledge of the candidate predictors (i.e., blinded)?
- The indices were apparent to the research team for death. Readmission data was collected blind to the candidate predictors. Readmission and death are regarded as objective outcome and the associated risk of bias is low.

5) Were candidate predictors part of the outcome (e.g. in panel or consensus diagnosis)?
- No

6) Time of outcome occurrence or summary of follow-up
- All patients were followed up. The outcome was 90 days after discharge.

Candidate predictors
1) Number and type of predictors (e.g. demographics, patient history, physical examination, additional testing, disease characteristics)
- The type of predictors is described. There were 22 candidate predictors.

2) Definition and methods for measurement of candidate predictors
- The methods for measuring each index are provided, as well as the definitions of those in the PEARL score. A data collection guide was provided to each hospital which included definitions and guidance on data collection.

3) Timing of predictor measurement of candidate predictors (e.g. at patients presentation, at diagnosis, at treatment initiation)
- Predictors were collected at the time of admission up to the point of the post-take ward round.

4) Were predictors assessed blinded for outcome, and for each other (if relevant)?
- The derivation cohort and external validation cohort were prospective. The internal validation cohort was performed retrospectively, but predictors were documented prior to the outcome (for example, eMRCD is collected within the COPD care bundle), and when extracting data reviewers were blind to the outcome. Collections of predictors were not blinded from each other, though the consequent risk of bias is low.

5) Handling of predictors in the modelling (e.g. Continuous, linear, non-linear transformations or categorised)
- Some predictors were categorised which is described.

Sample size
1) Number of participants and number of outcomes/events
2) Number of outcomes/events in relation to the number of candidate predictors (events per variable)
- 1+2) In the derivation cohort, internal validation and external validation cohorts there were 824, 802 and 791 patients. There were 22 candidate predictors in the derivation cohort, and 309 events, or 14. events per index. The power calculation for the derivation cohort and each individual validation cohort is described.

Missing data
1) Number of participants with any missing value (include predictors and outcomes)
2) Number of participants with missing data for each predictor
3) Handling of missing data (e.g. complete-case analysis, imputation, or other methods)

- 1+2+3) Missing data by participant and by index provided. Missing data rates were low. Multiple imputation was used, and the approach and number of datasets used described. Five datasets were used which is regarded as sufficient given the amount of missing data; complete-case analysis was performed.

Model development

1) Modeling method (e.g. logistic, survival, neural networks, or machine learning techniques)

- Logistic regression used; methods described.

2) Modeling assumptions satisfied

- Yes

3) Methods for selection of predictors for inclusion in multivariable modelling (e.g. all candidate predictors, pre-selection based on unadjusted association with the outcome)

- Predictors for inclusion selected based on literature search and clinical experience.

4) Method for selection of predictors during multivariable modeling (e.g. full model approach, backward or forward selection) and criteria used (e.g. p-value, Akaike Information Criterion)

- Backwards logistic regression used, criteria (p-values) described. The PEARL (full model) and PEARL (age continuous) were compared with the Akaike Information Criterion which showed no difference.

5) Shrinkage of predictor weights or regression coefficients (e.g. no shrinkage, uniform Shrinkage, penalized estimation)

- 5) Shrinkage refers to adjusting coefficients to protect against over-fitting and loss of discrimination in validation studies. Weightings were assigned to the PEARL score based on the regression coefficients from the derivation study. There are various strategies that have been suggested to improve the generalisability of prognostic tools. Some of these do not apply when there are multiple validation cohorts. We took the mean regression coefficient across all cohorts, and compared it to those in the derivation study. "Previous admission" was under scored compared to the eMRCD score. We adjusted "previous admissions" from a weighting of 2 to 3. This fits with previous research that suggests previous admissions is consistently one of the strongest predictors of readmission risk, and will maximise the generalisability of PEARL.

Model performance

1) Calibration (calibration plot, calibration slope, Hosmer-Lemeshow test) and Discrimination (C-statistic, D-statistic, log rank) measures with confidence intervals

- Hosmer-Lemeshow test provided. Actual and observed risks are shown in a calibration plot, and observed risk across all cohorts shown in table E3. Discrimination was calculated with AUROC curves (and confidence intervals provided).

2) Classification measures (e.g. sensitivity, specificity, predictive values, net reclassification improvement) and whether a priori cut points were used

- Sensitivity and specificity are provided, with PEARL scores used as cut-offs. Reclassification measures, such as net reclassification improvement, look at the value in adding a single predictor to a prediction model. No reclassification measures were performed.

Model evaluation

1) Methods used for testing model performance: development dataset only (random split of data, resampling methods, e.g. bootstrap or cross-validation, none) separate external validation (e.g. temporal, geographical, different setting, different investigators)

- Internal validation involved the same hospitals as the derivation cohort, but at a different time period (a form of temporal validation). External validation was performed at four hospitals. Hospitals were chosen for their differences, as described in the paper, to maximise generalisability. The research staff within external sites were not involved in the derivation or internal validation cohorts.

2) In case of poor validation, whether model was adjusted or updated (e.g. intercept recalibrated, predictor effects adjusted, or new predictors added)

- Not applicable

Results

1) Final and other multivariable models (e.g. basic, extended, simplified) presented, including predictor weights or regression coefficients, intercept, baseline survival, model performance measures (with standard or confidence Intervals)
2) Any alternative presentation of the prediction models. e.g. sum score, nomogram, score chart, predictions for specific risk subgroups with performance
3) Comparison of the distribution of predictors (including missing data) for development and validation datasets

- 1+2+3) Predictor weights and regression coefficients are given for the PEARL score. All models have AUROC calculated with confidence intervals. No subgroup analysis performed. Missing data rates for both all three cohorts was low.

Interpretation and discussion

1) Interpretation of presented models (confirmatory, if model useful for practice versus exploratory, is more research needed)

- The performance of PEARL is shown in three cohorts, with consistent risk stratification. Quantifying the impact of using PEARL requires further research.

2) Comparison with other studies, discussion of generalisability, strengths and limitations.

- Described in discussion
